# Anti-lipopolysaccharide egg yolk antibodies enhance the phagocytosis of mammalian phagocytes

**DOI:** 10.1242/bio.032821

**Published:** 2018-05-08

**Authors:** Xin Zhou, Siyuan Ma

**Affiliations:** Institute of Burn Research, State Key Laboratory of Trauma, Burns and Combined Injury, Third Military Medical University (Army Medical University), Chongqing 400038, China

**Keywords:** Egg yolk antibody, Anti-LPS IgY, THP-1, Mouse peritoneal macrophages, Phagocytosis

## Abstract

Macrophages play crucial roles in combatting infectious diseases by promoting inflammation and phagocytosis. The decline of macrophage phagocytic function will bring many serious consequences, including weakened pathogen clearance. As an avian antibody, immunoglobulin Y (IgY) has been widely used in preventing and treating infectious diseases, but whether it can enhance the phagocytic ability of mammalian macrophages in order to clear pathogens is still unknown. In this study, mouse peritoneal macrophages and THP-1 cells were cultured with anti-lipopolysaccharide (LPS) IgY *in vivo* or *in vitro*, respectively. Morphological observation, ELISA, fluorescence immunoassays and flow cytometry were used to study whether IgY could enhance phagocytosis of mammalian macrophages. It was found that without anti-LPS IgY, mouse peritoneal macrophages showed adherent growth with no differentiation and little pseudopod extension; but with anti-LPS IgY, peritoneal macrophages presented more significant characteristics in adherent growth, extension deformation and protruding pseudopods. With flow cytometry, the macrophages from mice injected with anti-LPS IgY exhibited a significantly higher percentage of phagocytosis and index (90.83±2.59% and 4.45±0.13 respectively) compared with phosphate buffered saline (PBS) groups (64.32±1.5%, and 2.36±0.11) and non-immunized groups (65.94%±1.4%, and 2.4±0.15). With phorbol-12-myristate-13-acetate (PMA)-induced THP-1 cells, similar results were found; the percentage and index were significantly higher, with larger body and more pseudopods, for THP-1 cells that were co-incubated with anti-LPS IgY (79.83±0.38% and 2.64±0.03), compared with cells that were co-incubated with PBS (68.07±0.52%, and 1.88±0.03) or non-immunized IgY (74.89±1.14% and 2.30±0.02). The results showed that anti-LPS IgY was effective in promoting the growth of macrophages, pseudopod extension and stronger phagocytic capacity. Our study indicated that anti-LPS IgY could enhance the phagocytic capacity of mammalian macrophages to internalize pathogens more effectively with larger body and more pseudopods. This may be important to encourage IgY to be used to prevent and treat infectious diseases.

## Abstract (precis)

**Summary:** Anti-lipopolysaccharide egg yolk antibodies can enhance the phagocytosis of mammalian phagocytes by promoting larger body and more pseudopods, and may be used as new healthcare or pharmaceutical products to prevent and treat infectious diseases.

## INTRODUCTION

Phagocytosis is a critical weapon of phagocytic cells; it helps them to hunt and engulf a variety of pathogens and foreign particles (Gray and Botelho, 2017). Macrophage, a kind of human phagocytic cell, with the effects of phagocytosis, immune information transmission, cooperation and phagocytosis of antigen processing, plays an important role in the outcome of inflammation (Guillemin and Brew, 2004). When the macrophages are activated, the phagocytic function of the macrophage is significantly enhanced, so as to assist the body in effectively removing harmful components. When the phagocytic function of macrophages is impaired, it brings a lot of serious consequences. Previous studies on the host defense function of human Cystic Fibrosis (CF) demonstrated that phagocytosis was significantly reduced in pulmonary phagocytes from pediatric CF lungs (Alexis et al., 2006).

Specific immunoglobulin Y (IgY) harvested from avians with high yield and low cost, could be used to prevent and treat infectious diseases by removing pathogenic microorganisms such as *Pseudomonas aeruginosa*, *Helicobacter pylori*, rotavirus, influenza virus and *Escherichia coli* (Rahman et al., 2013; Yang et al., 2012; Zhen et al., 2011; Thibodeau et al., 2017; Hong et al., 2018; Thomsen et al.; Shi et al., 2017; Pizarro-Guajardo et al., 2017). Therefore, IgY may have a positive role in preventing and controlling infectious diseases by enhancing the phagocytic function of mammalian macrophages to remove pathogenic bacteria effectively. This study will mainly focus on whether anti-LPS IgY could enhance the phagocytosis of mammalian macrophages.

Lipopolysaccharide (LPS), as a component of the cell wall of gram negative bacteria, is an important pathogenic factor. It is recognized as the most potent microbial mediator that is implicated in the pathogenesis of sepsis and septic shock (Opal, 2007, 2010; Bottiroli et al., 2017). In our previous works, we used LPS as the immunogen, and when we prepared the anti-LPS IgY, we found that the anti-LPS IgY (which was harvested as a polyclonal antibody from the egg yolks of immunized hens) may be a possible tool for the prevention and treatment of LPS injuries (Ma and Zhang, 2010). Macrophages have the following functions: phagocytosis, antigen presentation and secretion of biologically active substances (Sóñora et al., 2017; Dale et al., 2008). Studies have found that the phagocytosis of macrophages is important in preventing and controlling infections from pathogenic microorganisms (Uribe-Querol and Rosales, 2017; Dallenga et al., 2017). Previous studies have suggested that IgY could enhance the internalization of corresponding pathogenic bacteria. Anti-*Pseudomonas aeruginosa* IgY antibodies promote specific bacterial aggregation and internalization in polymorphonuclear neutrophils (Thomsen et al., 2016). We wanted to find out whether anti-LPS IgY plays a positive role in the prevention and control of infectious diseases in mammals and humans by enhancing phagocytosis.

THP-1 (human acute monocytic leukemia cell line) cells, which can be induced by many agents to become macrophage-like cells that are capable of phagocytosis (Park et al., 2007), have been widely used in inflammatory diseases studies, and phorbol-12-myristate-13-acetate (PMA) is often used as an inducing agent. In this study, we used THP-1 cells and mouse peritoneal macrophages as targets to observe *in vitro* and *in vivo*, respectively, using fluorescent immunoassays and flow cytometry to determine the effect of anti-LPS IgY on the growth and morphology of the two kinds of cells. We were also observing the effects of IgY on the nonspecific phagocytosis of fluorescent beads, so as to evaluate the effect of anti-LPS IgY on the phagocytosis of macrophages.

## RESULTS

### Preferred THP-1 cell concentration, culture time and PMA concentration

**Fig. 1.**
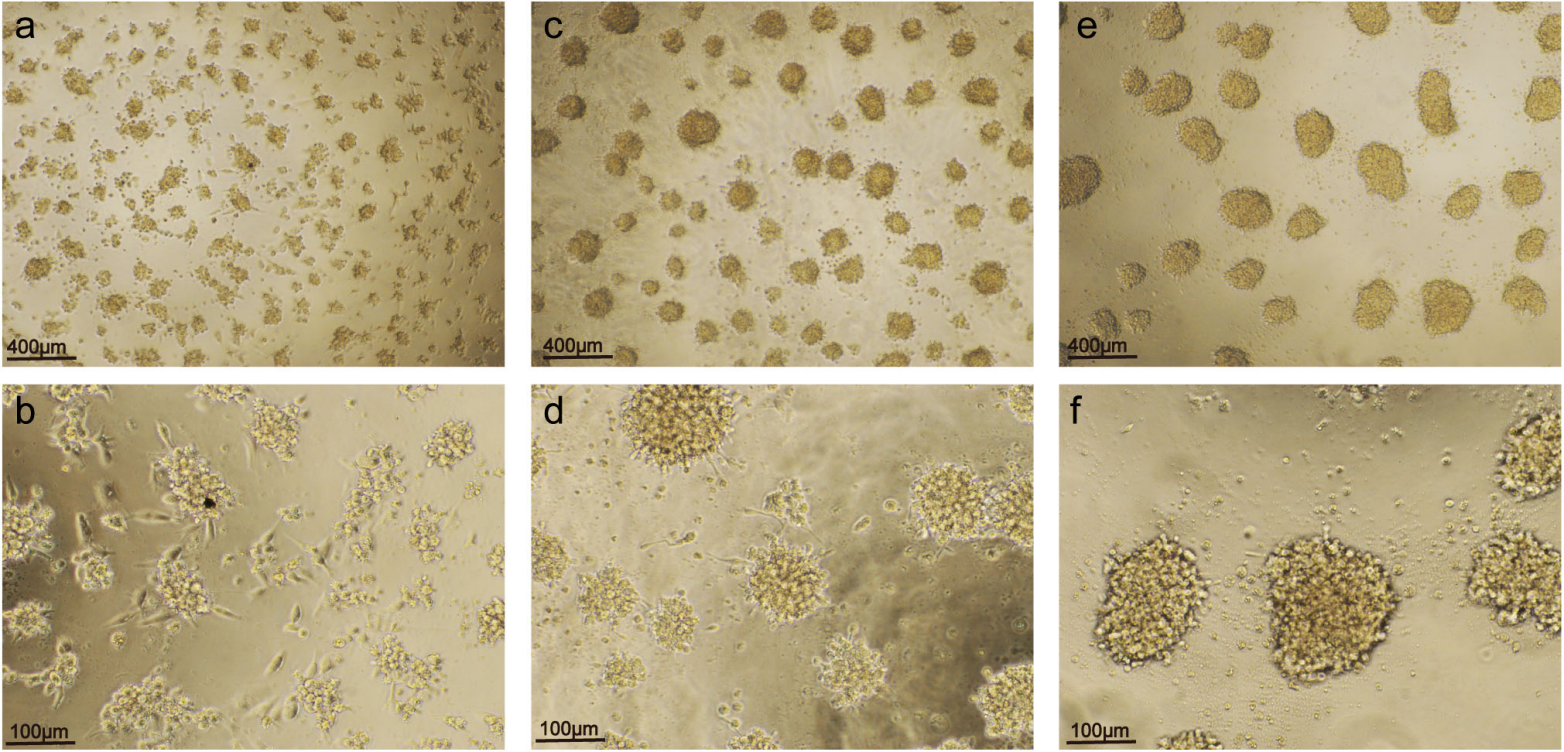
**Effects of cell density on the differentiation of the THP-1 cells to macrophage-like cells induced by PMA.** (A,B) When the cell density was 0.25×10^6^/ml, the THP-1 cells differentiated well, and represented macrophage-like growth. (C,D) When the cell density was 0.5×10^6^/ml, the THP-1 cells were clustered into large groups and few cells were free to differentiate into macrophage-like cells. (E,F) When the cell density was 1.0×10^6^/ml, the THP-1 cells were clustered into much larger groups and floated in the cell culture solution and no cells were free to differentiate into macrophage-like cells. (A) 0.25×10^6^/ml (×40). (B) 0.25×10^6^/ml (×100). (C) 0.5×10^6^/ml (×40). (D) 0.5×10^6^/ml (×100). (E) 1.0×10^6^/ml (×40). (F) 1.0×10^6^/ml (×100).

In the presence of PMA, THP-1 cells were adherent and aggregated. When the cell density decreased to 0.25×10^6^/ml, PMA induced stimulation of adherent cell aggregation, but evenly distributed the formation of large clumps of cells. Cell differentiation was significant and showed deformation and growth of pseudopodia as macrophage-like cells (Fig. 1).

**Fig. 2.**
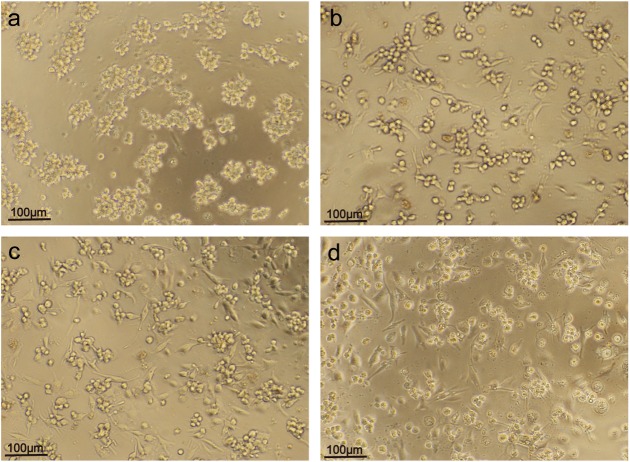
**Effects of time on differentiation of THP-1 cells to macrophage-like cells induced by PMA.** (A) After 24 h, the THP-1 cell induced by PMA started to adhere, cluster and very few cells presented deformation. (B) After 48 h, most of the THP-1 cells were macrophage-like, but the cells were still small. (C) After 72 h, the THP-1 cells were macrophage-like and had grown much larger. (D) After 96 h, the state of the THP-1 cells was similar to at 72 h. (A) 24 h (×100). (B) 48 h (×100). (C) 72 h (×100). (D) 96 h (×100).

With more time, THP-1 cells gradually differentiated in suspension from cells adherent to macrophage-like cells with pseudopodia under the effect of PMA. Cell aggregations in the appropriate concentration of PMA for 72 h showed a clear pseudopodia and deformation of macrophage-like growth; the cell body increased significantly. (Fig. 2).

**Fig. 3.**
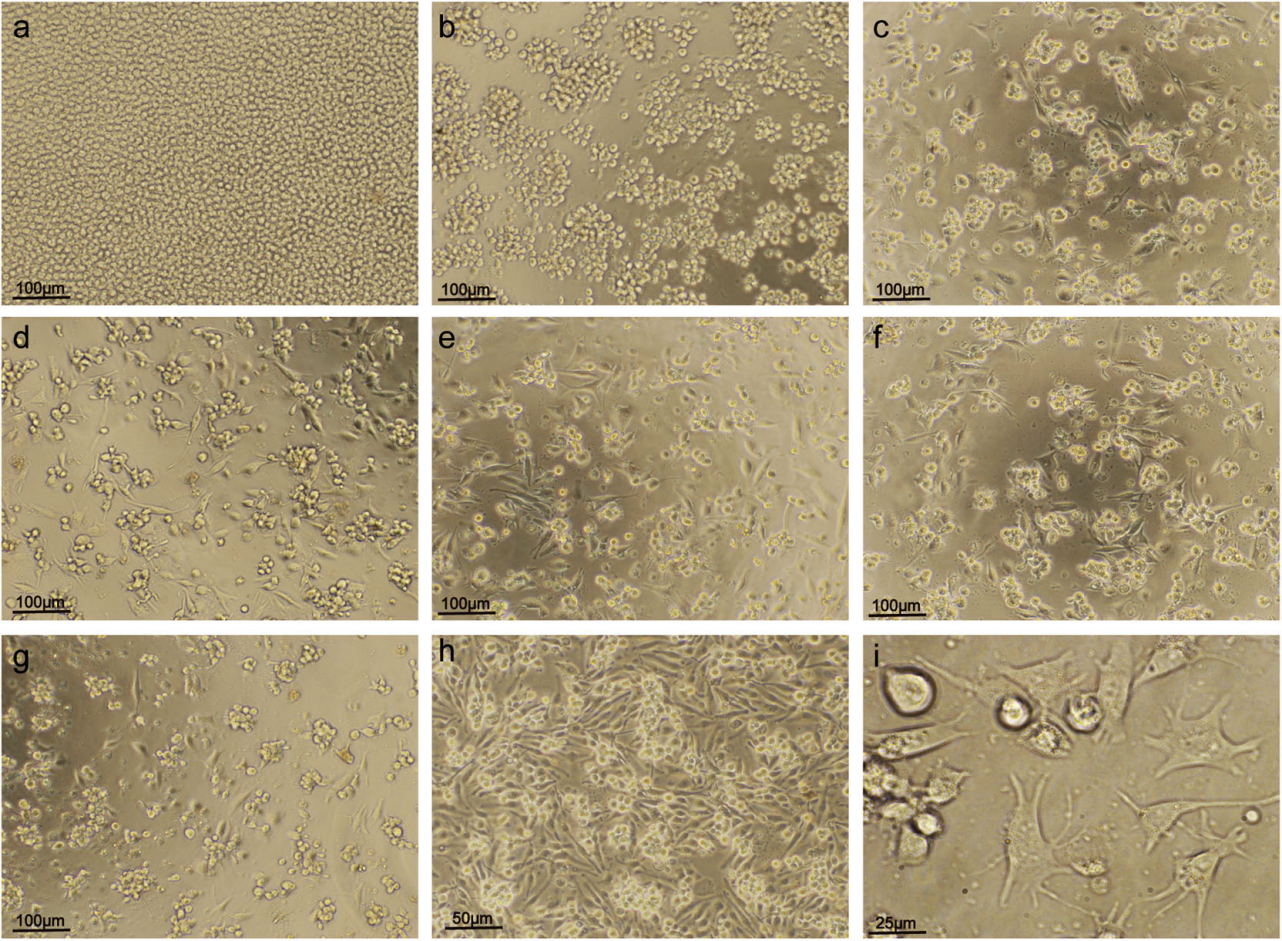
**Effects of PMA concentration on differentiation of THP-1 cells to macrophage-like cells induced by PMA.** (A) When no PMA was added to the THP-1 cells, the THP-1 cells floated in the cell culture solution with significant proliferation. (B) When the PMA concentration increased to 2 ng/ml, a few of the THP-1 cells adhered and differentiated into macrophage-like cells. (C-I) When the PMA concentration was equal to or more than 5 ng/ml, most of THP-1 cells adhered and differentiated into macrophage-like cells. (A) 0 (×100). (B) 2 ng/ml (×100). (C) 5 ng/ml (×100). (D) 10 ng/ml (×100). (E) 20 ng/ml (×100). (F) 50 ng/ml (×100). (G) 100 ng/ml (×100). (H) 10 ng/ml (×200). (I) 10 ng/ml (×400).

**Fig. 4.**
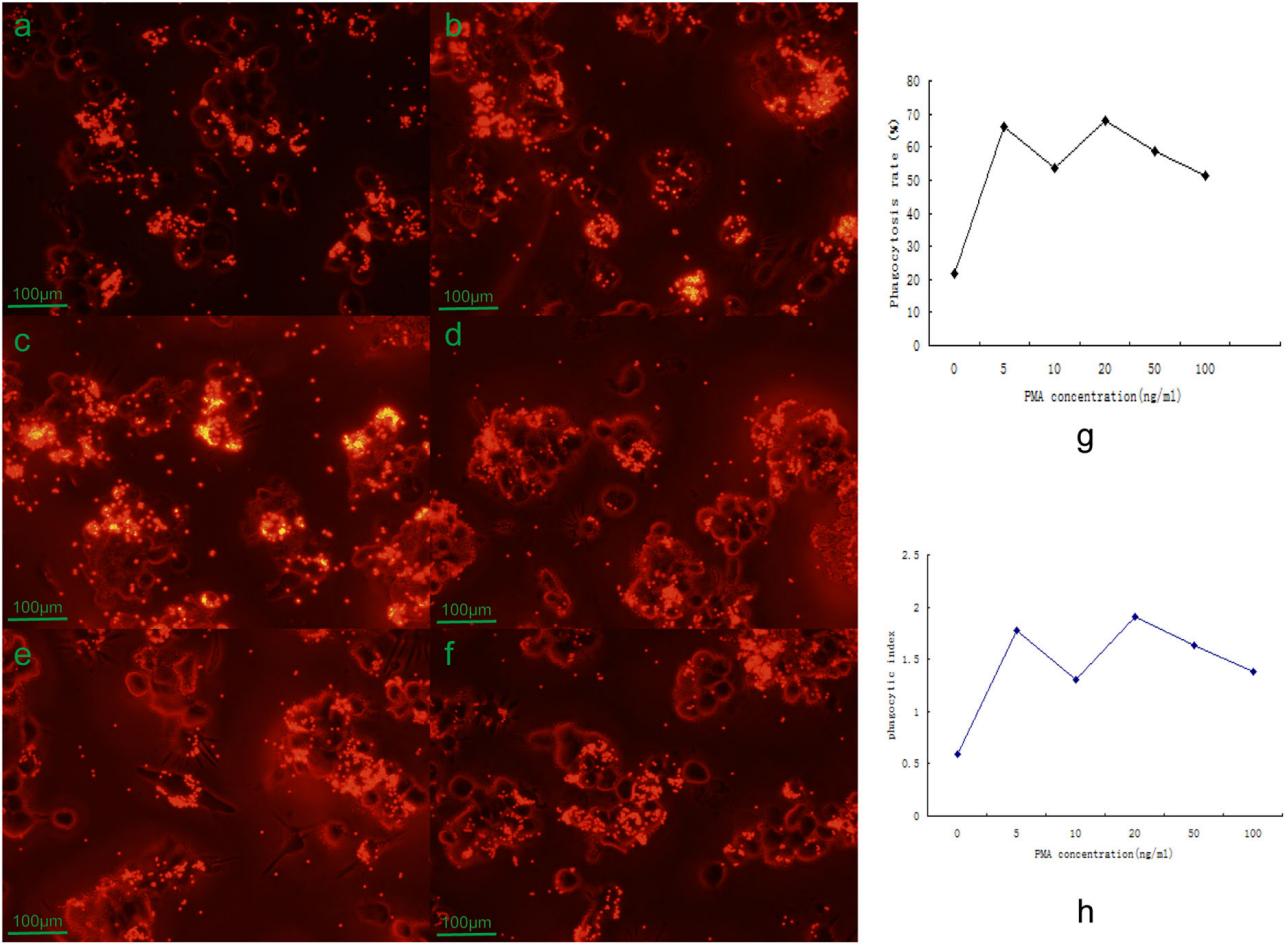
**Effect of PMA concentration on the ability of the THP-1 cells to phagocytize the fluorescent beads.** (A) When the PMA concentration was 2 ng/ml, some THP-1 cells phagocytized fluorescent beads. (B) When the PMA concentration was 5 ng/ml, the number of THP-1 cells that phagocytized the beads was greatly increased; the phagocytosis rate and phagocytosis index also increased. (C-F) When the concentration of PMA was 5 ng/ml or over, in measurements of 10 ng/ml (C), 20 ng/ml (D), 50 ng/ml (E) and 100 ng/ml (F), the results were similar in that most of the THP-1 cells phagocytized the beads and the phagocytosis index was similar; there was no significant difference among them (*P*>0.1). (A) 2 ng/ml (×100). (B) 5 ng/ml (×100). (C) 10 ng/ml (×100). (D) 20 ng/ml (×100). (E) 50 ng/ml (×100). (F) 100 ng/ml (×100). (G) Phagocytosis rates. (H) Phagocytosis index.

When cultured for 72 h, THP-1 cells showed adherent deformation and grew obvious pseudopods with PMA concentration from 5 ng/ml to 100 ng/ml, but at the 10 ng/ml PMA, the larger body and more pseudopods were observed (Fig. 3). The internalized fluorescent microspheres by PMA-induced THP-1 cells were also observed using a fluorescent microscope and flow cytometry (Fig. 4).

**Fig. 5.**
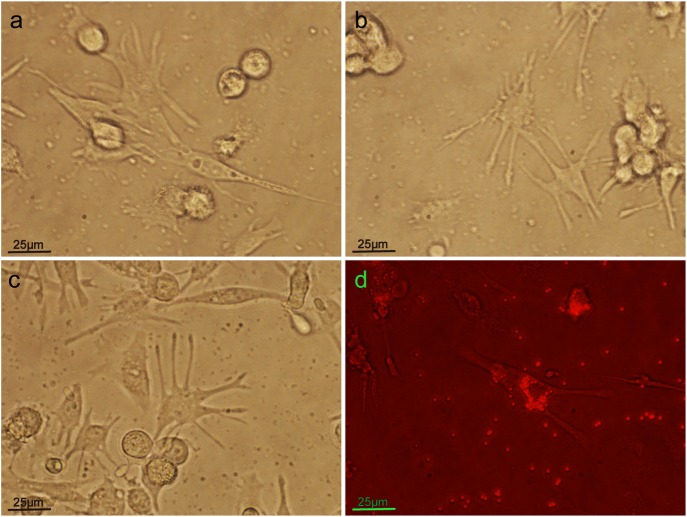
**Differentiated THP-1 cells with many long pseudopodia and macrophage-like cell shape after implementing induction conditions: a lower concentration of PMA(10 ng/ml), a shorter time (72 h) and a lower density of THP-1 (0.25×106/ml).** (A-C) Morphology of pTHP-1 cells. (D) Phagocytosis of fluorescent beads by pTHP-1 cells.

Finally, according to the above experimental results, we selected reasonable conditions to differentiate THP-1 cells into cells with more long pseudopodia and macrophage-like cell shapes (Fig. 5) under a lower concentration of PMA (10 ng/ml), in a shorter time (72 h) and with a lower density of THP-1 (0.25×106/ml).

### Morphology of mouse peritoneal macrophage

**Fig. 6.**
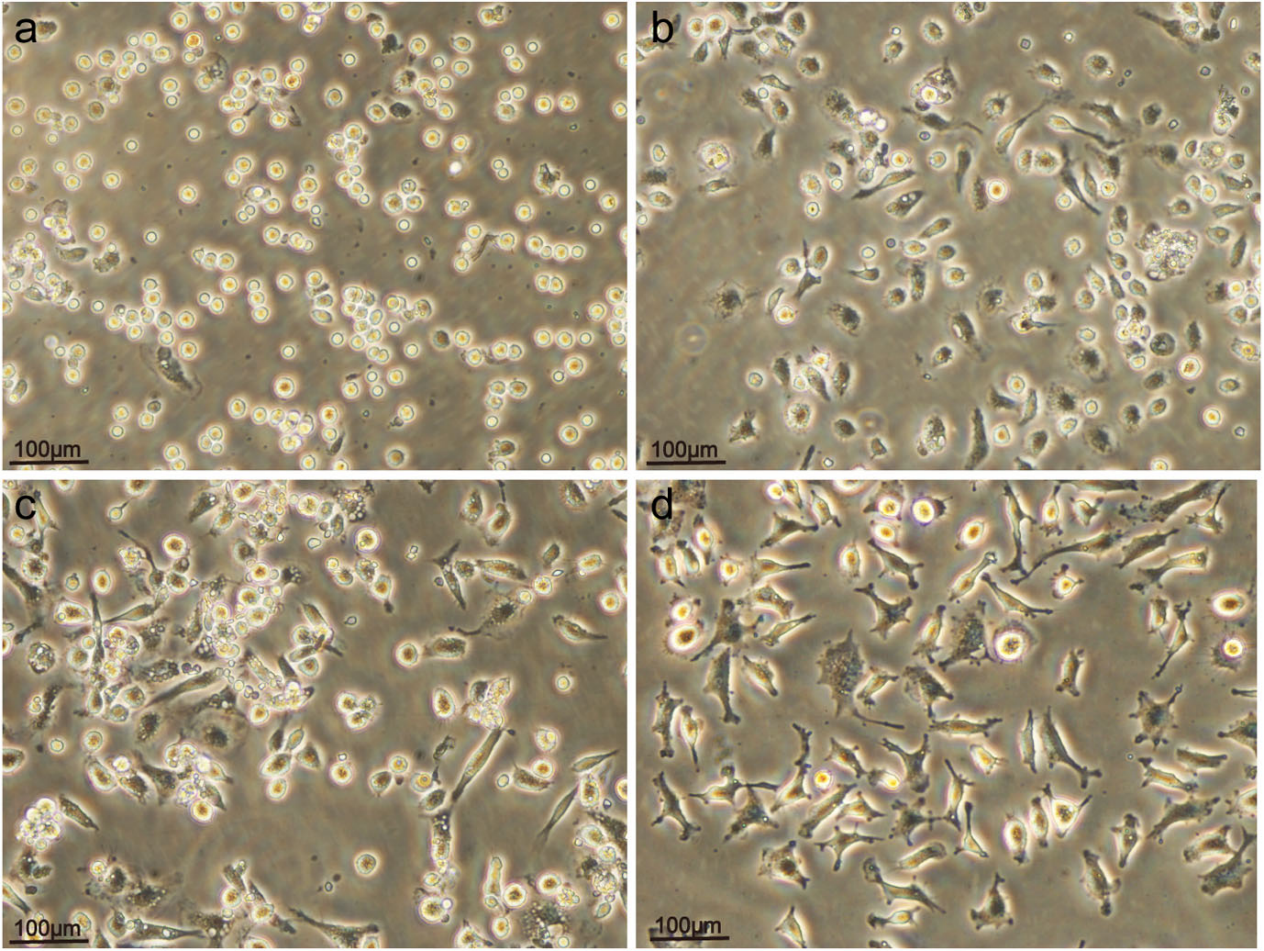
**Peritoneal macrophages isolated from mice that received peritoneal injections of different agents.** The peritoneal macrophages were harvested from Kunming mice. (A) A control group. (B) A group that received a peritoneal injection of PBS. (C) A group that received a peritoneal injection of non-immunized IgY. (D) A group that received a peritoneal injection of anti-LPS IgY. All mice were injected with their respective agents once per day for 7 days. The cell morphology was then observed under a microscope (×100).

After 2 h of cultivation *in vitro*, the normal mouse peritoneal macrophages showed adherent growth but less deformation and pseudopod extension. However, the macrophages collected from mice that received intraperitoneal injections of PBS, non-immunized IgY and anti-LPS IgY exhibited obviously adherent growth and extensional deformation, which was accompanied by protruding pseudopods in large and various shapes (Fig. 6).

**Fig. 7.**
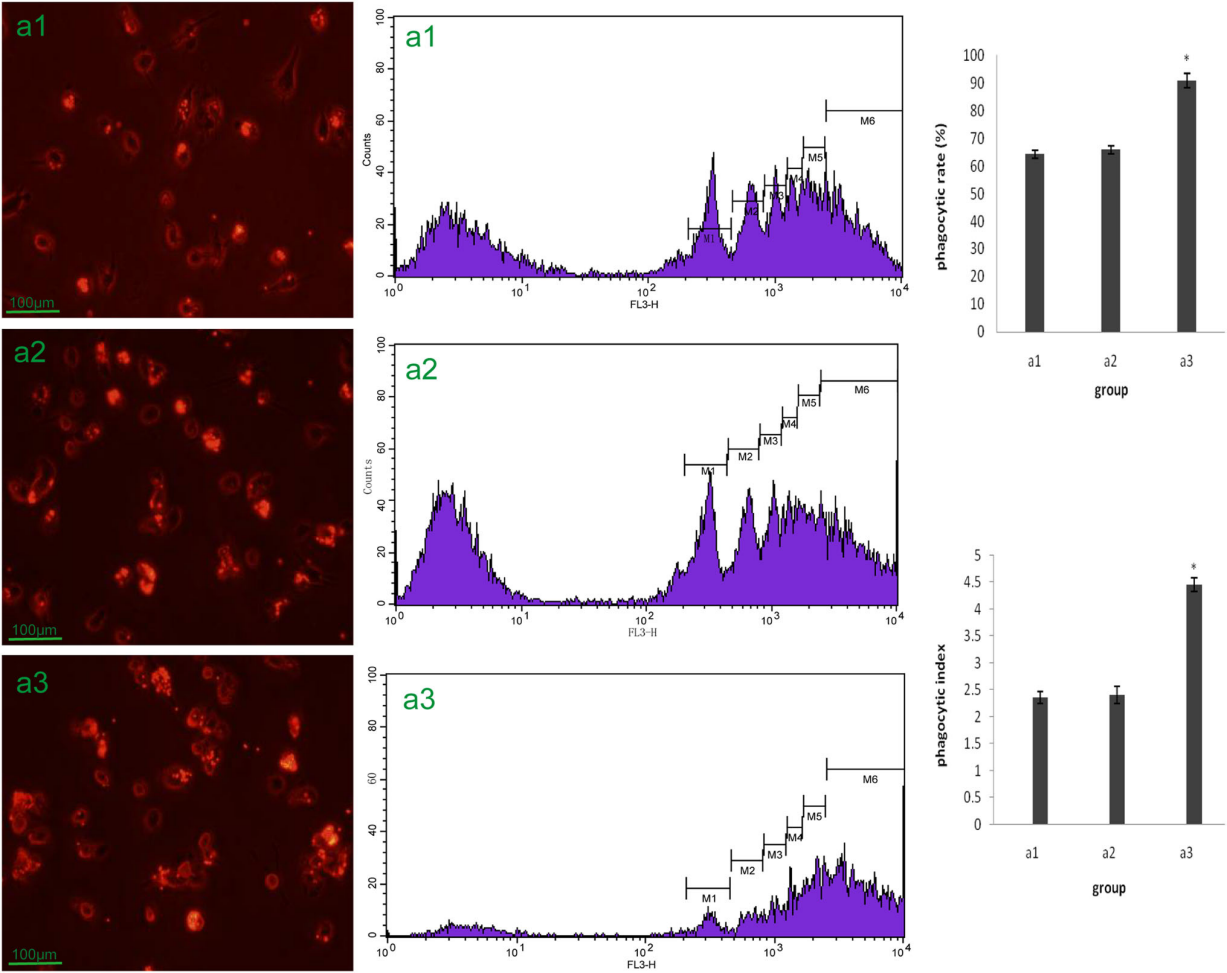
**Phagocytosis of peritoneal macrophages from Kunming mice.** Mice received peritoneal injections of PBS, non-immunized IgY or anti-LPS IgY once per day for 7 days. The peritoneal macrophages from the different mice were incubated with fluorescent beads for 2 h *in vitro*. The phagocytic activity of the macrophage cells were determined by the number of phagocytized fluorescent beads, which was observed under a fluorescent microscope. (A1) Mice injected with PBS. (A2) Mice injected with non-immunized IgY. (A3) Mice injected with anti-LPS IgY (*Kruskal–Wallis Test *P*=0.000).

After the adherent mouse peritoneal macrophages were incubated with fluorescent beads for 2 h, the phagocytosis of the macrophages was determined using fluorescent microscopy and flow cytometry. Although all of the macrophages phagocytized a large number of red fluorescent beads, as shown in the results obtained with the fluorescent microscope, there were obvious differences among the cells obtained from the three groups of mice. The macrophages harvested from anti-LPS IgY injected mice (i.p. anti-LPS IgY, 16 mg for 7 days) exhibited a higher phagocytosis percentage and index than cells from PBS injected mice and non-immunized IgY injected mice (Fig. 7).

### Phagocytosis of PMA-induced THP-1 cells

**Fig. 8.**
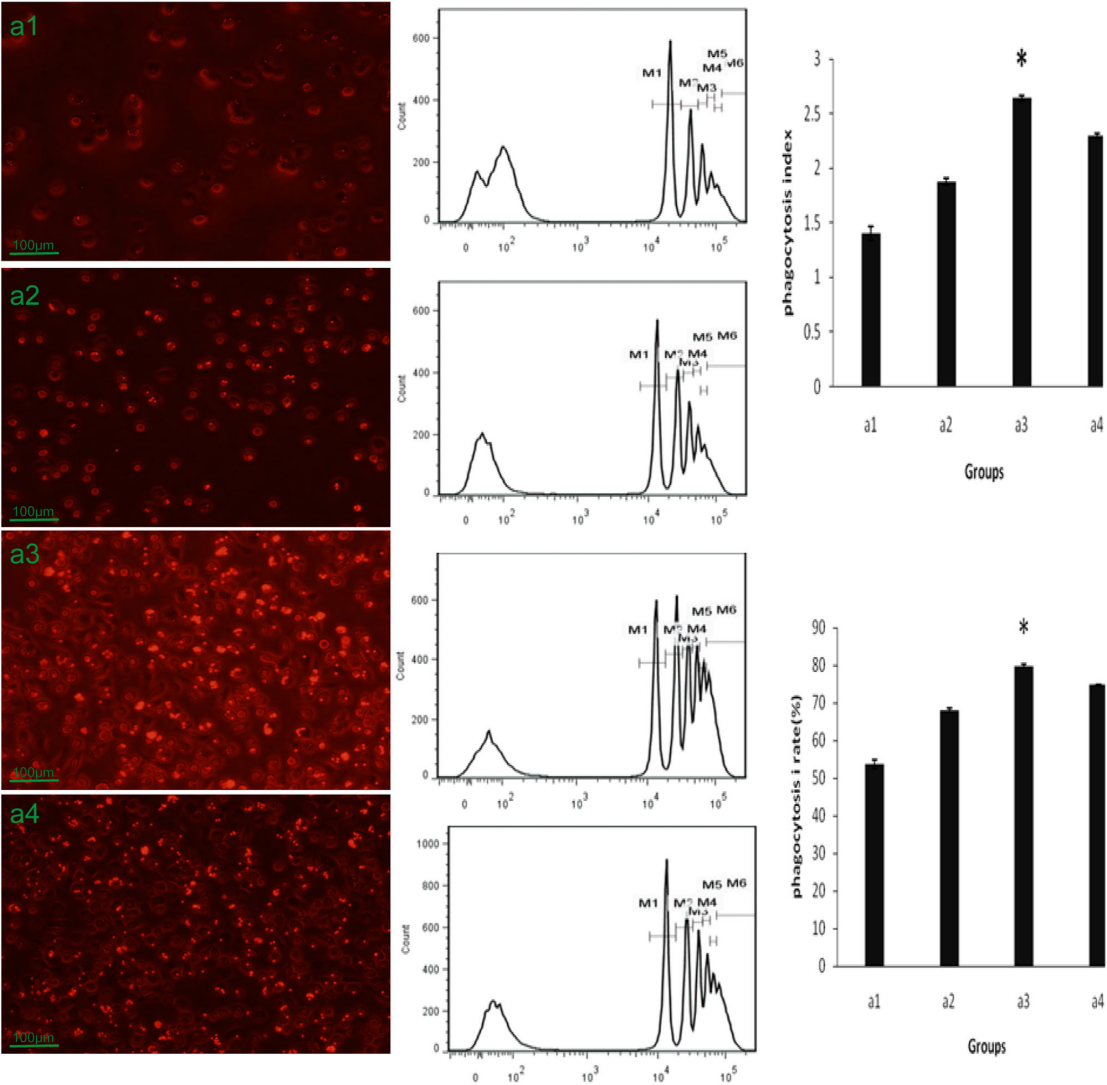
**Effect of anti-LPS IgY on the phagocytosis of PMA-induced THP-1 cells.** After their induction by PMA for 72 h, the pTHP-1 cells were incubated with anti-LPS IgY or non-immunized IgY for an additional 24 h. The phagocytic activity of the pTHP-1 cells was then determined by the number of phagocytized fluorescent beads. pTHP-1 cells that were cultured without IgY served as the control. (A1) pTHP-1 cells. (A2) pTHP-1 cells incubated with PBS. (A3) pTHP-1 cells incubated with anti-LPS IgY. (A4) pTHP-1 cells incubated with non-immunized IgY (*Kruskal–Wallis Test *P*=0.016).

After cell culture, we observed that the PMA-induced THP-1 cells engulfed a large number of fluorescent particles and thus emitted a red fluorescence under the fluorescent microscope. The phagocytic capacity of the PMA-induced THP-1 cells was enhanced significantly by anti-LPS IgY, with the percentage of cells which engulfed beads increased and the average number of engulfed particles by a single cell also increased. However, the cells treated with non-immunized IgY did not exhibit the same phagocytic ability as those treated with anti-LPS IgY. Flow cytometry was used to confirm the above results. Without PMA treatment, few THP-1 cells engulfed fluorescent beads; after PMA treatment, the phagocytosis percentage and phagocytosis index increased. When incubated with anti-LPS IgY for 24 h, the phagocytosis percentage and phagocytosis index increased significantly. However, the phagocytosis percentage and index did not increase after co-incubation with non-immunized IgY (Fig. 8).

## DISCUSSION

THP-1 cells, which are capable of macrophage phagocytosis and secretion of inflammatory mediators, are often used to study the function of human macrophages. PMA is one of the most commonly used inducers. However, when PMA was used to induce THP-1 cells to differentiate into macrophage-like cells, there was a lack of relative standards for specific induction conditions, and there were too many arbitrary factors. For example, the induction dose of PMA could reach 400 ng/ml from 10 ng/ml (Chen and Ross, 2004; Gatto et al., 2017; Kong et al., 2016; Bener et al., 2016). The induction time of PMA ranged from 24 h to 72 h (Park et al., 2007; Zhou et al., 2010; Yang et al., 2008). Studies have shown that when using PMA to induce THP-1 differentiation, the different concentrations of PMA can cause significant differences in the gene expression of THP-1 cells and affect the biological function of cells (Zhang et al., 2006). The key factors related to macrophage phagocytic function were the morphological differentiation, the size and the number of macrophages. Therefore, in order to find the appropriate induction conditions including cell density, induction concentration time and PMA concentration to induce THP-1 cells into macrophage-like cells, we observed the morphological changes and cell adhesion and phagocytosis of fluorescent beads. Finally, according to these results, combined with the available literature, we selected the appropriate conditions to differentiate THP-1 cells into macrophage-like cells with many long pseudopodia (Fig. 5), under lower concentration of PMA (10 ng/ml), within a shorter time (72 h) and with a lower density of THP-1 (0.25×10^6^/ml), according the needs of our study.

In this study, the phagocytic function of PMA-induced THP-1 cells was demonstrated to be significantly enhanced after the cells were co-incubated with anti-LPS IgY for 24 h. The phagocytic rate and the phagocytic index of these cells were significantly increased. These results suggest that anti-LPS IgY may possess the ability to enhance the phagocytic activity of macrophages. Our previous studies have shown that IgY could improve the survival of LPS-challenged mice significantly (Ma and Zhang, 2011). Enhancing the phagocytic activity of macrophages might be one of the mechanisms of anti-LPS IgY to attenuate LPS injuries and improve the survival of mice challenged by LPS. Furthermore, we performed *in vivo* studies that confirmed these results. The intraperitoneal injection of anti-LPS IgY to healthy Kunming mice for seven consecutive days significantly enhanced the phagocytic activity of the peritoneal macrophages of these mice. As we know, severe trauma and infections such as bacteremia decrease the phagocytic activity of monocytes and macrophages. We theorized that anti-LPS IgY could help improve the treatment of serious infections, and even sepsis, by enhancing the phagocytic ability of macrophages.

Our study showed that when incubating anti-LPS IgY and PMA-induced THP-1 cells pTHP-1 cells for 24 h, phagocytic rate and phagocytic index of pTHP-1 cells were significantly increased. It suggested that anti-LPS IgY could enhance the phagocytic activity of macrophage function. The results were also confirmed by subsequent animal experiments. We found that the phagocytic capacity of peritoneal macrophages was significantly enhanced after 7 days of intraperitoneal injection of anti-LPS IgY in healthy Kunming mice.

How did IgY enhance the phagocytic function in mammalian macrophages? Previously, it was thought that chicken IgY might be converted into a different substance in the body that played a role in immune opsonization in the mammalian immune system. Lee et al. (2002) reported that the interaction of specific IgY with antigens on the bacterial surface could lead to changes in the electron cloud and thus the charge of the bacterial wall. These changes might help the phagocytic cell access, adhere to, catch and engulf bacteria (Lee et al., 2002). However, in our study, we did not co-incubate the IgY and the fluorescent beads, and we used a new culture solution without IgY after the incubation of the pTHP-1 cells with IgY in order to eliminate the chance that the fluorescent beads were co-incubated with IgY. Our results indicated that purified anti-LPS IgY still could act on the PMA-induced THP-1 cells directly, enhancing the phagocytosis of macrophages by growing larger body and more pseudopods.

The phagocytic function of pTHP-1 cells and mouse peritoneal macrophages were enhanced by anti-LPS IgY, with phagocytic rate and phagocytic index significantly increased. However, there were no similar effects for the non-immunized IgY. These results may have been caused by the differences in structure between anti-LPS IgY and non-immunized IgY; the specific reasons need further study.

Above all, our study showed that anti-LPS IgY could enhance the phagocytosis of mammalian macrophages directly by creating larger body and more pseudopods. Our findings suggest that IgY could be used to develop health care or pharmaceutical products as it is a new agent that enhances the macrophages’ ability to phagocytize microorganisms, preventing and treating infectious diseases, with a number of advantages, such as its plentiful source, low cost and cruelty-free acquisition.

## MATERIALS AND METHODS

### THP-1 culture and optimization of PMA concentration

THP-1 monocytic cells (ATCC, TIB202) (2.5×10^5^/ml, 5×10^5^/ml, and 10×10^5^/ml) were differentiated into macrophages in a Costar 96-well plate containing 100 µl of RPMI 1640 medium supplemented with 10% FCS and PMA (Sigma-Aldrich catalog No. P-8139) at 37°C in 5% CO_2_.

The amount of PMA was set to 10 ng/ml, and the culture time was set to 72 h to study the THP-1 cell densities of 2.5×10^5^/ml, 5×10^5^/ml, and 10×10^5^/ml. THP-1 cell density of 2.5×10^5^/ml and PMA concentration of 10 ng/ml was used for different culture times (24, 48, 72 and 96 h). THP-1 cell density of 2.5×10^5^/ml and culture time of 72 h were used with different PMA concentrations: 0, 2, 5, 10, 20, 50 and 100 ng/ml.

After cell culture, fluorescent beads (diameter of 2 µm; Sigma-Aldrich catalog no. L3030) were added at a bead to cell ratio of 20:1 and mixed. After 2 h of incubation at 37°C and 5% CO_2_, the supernatant was discarded, and the cells were washed with cool phosphate buffered saline (PBS) three times to remove the fluorescent beads that were not swallowed.

After a 60 s digestion by trypsin, RPMI 1640 medium supplemented with 10% fresh calf serum was added to terminate the digestion reaction. Cells were collected in sterile EP tubes by centrifugation (3000 rpm, 10 min) and then used in flow cytometry analysis.

The phagocytic rate (100%) is the number of cells that swallowed beads, divided by the number of total cells. The phagocytic index is the number of fluorescent beads that were swallowed, divided by the number of total cells. The number of fluorescent beads that were swallowed was calculated using the following formula: number of swallowed fluorescent beads=number of cells that swallowed one bead×1+number of cells that swallowed two beads×2+number of cells that swallowed three beads×3+number of cells that swallowed four beads×4+number of cells that swallowed five beads×5+number of cells swallowed six beads×6.

For the morphology study, the cells were washed with cool PBS and then fixed with 4% formalin for 10 min, then washed with PBS to remove all of the beads that were not swallowed and then observed and photographed under the inverted fluorescence microscope.

### Harvest of mouse peritoneal macrophages

Kunming mice (8-12 weeks of age, 30g±0.5 g in weight), provided by the Laboratory Animal Center at Third Military Medical University (Chongqing, China), were housed in cages in a standard animal room. The experimental procedure was approved by the Ethical Committee of Southwest Hospital at Third Military Medical University. Mouse peritoneal macrophages (Mφs) were collected (in sterile PBS) from the peritoneal cavity of unstimulated mice. The cells were washed, centrifuged (4°C, 1100 g, 10 min), re-suspended in RPMI-1640 supplemented with 10% fetal bovine serum (FBS), penicillin G (100 U/ml), streptomycin (100 µg/ml) and amphotericin B concentration(1.2 µg/ml), and plated on six-well culture plates at a density of 5×10^5^ cells/well. After being incubated for 2 h at 37°C in 5% CO_2_ air, the adherent Mφs were washed three times with RPMI-1640 to remove all of the non-adherent cells.

### Morphology study of THP-1 and murine macrophage after IgY treatment

Twelve Kunming mice were divided into four groups: normal group, i.p. PBS injection group, i.p. non-immunized IgY injection group and i.p. anti-LPS IgY injection group. According to our previous study (Zhang et al., 2006), we found that when the ratio of LPS to IgY was 1:16, anti-LPS IgY had the best treatment effect on burned mice suffering intestinal endotoxemia. The mice in the i.p. PBS injection group were injected with 1 ml of PBS once every day. The mice in the non-immunized IgY intraperitoneal injection group and anti-LPS IgY intraperitoneal injection group were injected with 16 mg of non-immunized IgY and 16 mg of anti-LPS IgY, respectively, every day. Seven days later, all of the mice were sacrificed. The Mφs cells were collected and cultured.

THP-1 cells at a cell density of 2.5×10^5^/ml (2 ml/well) were cultured in six-well tissue culture plates for 48 h at 37°C and 5% CO_2_ with 10 ng/ml PMA. Then, non-immunized IgY (16 µg/ml) and anti-LPS IgY (16 µg/ml) were added to the respective wells of each plate and observed at 24 h, 48 h and 72 h.

For morphology study, the Mφs cells were washed with cool PBS and fixed with 4% formalin for 10 min, then washed with PBS to remove all of the beads that were not swallowed. Finally, they were observed using an inverted fluorescent microscope.

### Phagocytosis study of THP-1 and murine macrophage after IgY treatment

THP-1 cells at a cell density of 2.5×10^5^/ml (2 ml/well) were cultured in six-well tissue culture plates for 48 h at 37°C and 5% CO_2_ with 10 ng/ml PMA. Then, non-immunized IgY (16 µg/ml) and anti-LPS IgY (16 µg/ml) were added to the respective wells of each plate.

After cell culture, fluorescent beads were added at a bead to cell ratio of 20:1 and mixed, and then incubated at 37°C and 5% CO_2_ for another 2 h. The supernatant was discarded and cells were washed with cool PBS three times to remove the fluorescent beads that were not swallowed. Finally, they were digested by trypsin and collected for flow cytometry analysis after being photographed under the microscope.

### Statistical analysis

SPSS 16.0 was used for statistical analysis. The Kruskal–Wallis Test was used to analyze the different sets of results.
